# Puberty timing and relative age as predictors of physical activity discontinuation during adolescence

**DOI:** 10.1038/s41598-023-40882-3

**Published:** 2023-08-23

**Authors:** François Gallant, Jeff J. Hebert, Véronique Thibault, Saïd Mekari, Catherine M. Sabiston, Mathieu Bélanger

**Affiliations:** 1 Département de Médecine de Famille et Médecine d’urgence, Université de Sherbrooke, Sherbrooke, QC J1K 2R1 Canada; 2 Centre de Formation Médicale du Nouveau-Brunswick, Moncton, NB E1A 7R1 Canada; 3 Faculty of Kinesiology, University of New Brunswick, Fredericton, NB E3B 5A3 Canada; 4 College of Science, Health, Engineering, and Education, Murdoch University, Murdoch, WA 6150 Australia; 5 Faculty of Kinesiology and Physical Education, University of Toronto, Toronto, ON M5S 2W6 Canada; 6 Vitalité Health Network, Moncton, NB E1C 2Z3 Canada

**Keywords:** Paediatrics, Epidemiology

## Abstract

Among same-age adolescents, those who enter puberty relatively later and those who are relatively younger (e.g., born later in the year) might be at greater risk of physical activity discontinuation. This study aimed to (1) describe gender-specific discontinuation, re-engagement, and uptake rates in various types of physical activities from the age of 11 to 17 years, and (2) assess puberty timing and relative age as predictors of discontinuation from organized, unorganized, individual, and group-based physical activities. Longitudinal data from 781 (56% girls, age 10–13 years at study baseline) Canadian participants who self-reported puberty status, birthdate, and involvement in 36 physical activities every four months from 2011 to 2018 was analyzed. The incidence of discontinuation, re-engagement, and uptake in organized/unorganized and individual/group activities from grade 6 until grade 12 was described and Cox proportional hazard models were used to estimate associations of puberty timing and relative age with organized/unorganized and individual/group activity discontinuation. Results demonstrate that individual and unorganized activities are maintained longer than group-based and organized activities. Girls who started puberty earlier were more likely to discontinue organized activities than girls with average-puberty timing [Hazard ratio (HR) (95% confidence interval (CI)) 1.68 (1.05–2.69)]. Compared to boys born in the 4th quarter of the year, boys born in the 2nd quarter of the year were less likely to discontinue organized [HR (95% CI) 0.41 (0.23–0.74)], unorganized [HR (95% CI) 0.33 (0.16–0.70)], group [HR (95% CI) 0.58 (0.34–0.98)], and individual activities[HR (95% CI) 0.46 (0.23–0.91)], and boys born in the 3rd quarter were less likely to discontinue unorganized activities[HR (95% CI) 0.41 (0.19–0.88)]. This study illustrates the patterns of physical activity participation throughout adolescence. However, the generalizability of findings may be limited due to participant representation.

## Introduction

Childhood and adolescence are marked by large declines in physical activity participation. By the age of 10 years, physical activity levels typically start to decrease at an average rate of 7% annually^1^. Although participation proportions change at different rates across various types of physical activity^2,3^, evidence suggests that adolescents are more likely to sustain participation in individual physical activities than group-based activities^2^. Beyond activity type, it is also important to describe longitudinal participation in physical activities from different contexts. For example, organized (e.g., structured activities requiring a coach/instructor and payment) and unorganized (e.g., unstructured activities without a coach/instructor and limited rules) forms of physical activity may have different probabilities of being sustained over time^4–6^ given unique barriers such as opportunity, availability, and cost^7^. Yet, sustainability and change in physical activity participation is not well-understood and no recent studies have been published^2,8,9^, existing evidence is limited by relatively short follow-up periods (e.g., 2 or 3 time points covering 2 to 4 years)^3,10,11^, and only a few physical activity types and/or contexts have been examined^9–11^. Furthermore, none of these studies identify predictors of sustainability or change in physical activity participation. It is critical to understand the types of physical activities that are the most likely to be maintained and when participation in specific physical activities is most likely to be discontinued to help inform and guide intervention efforts aimed at enhancing physical activity participation throughout adolescence.

One potential predictor of changes in physical activity participation during adolescence is the onset of puberty^12,13^. Puberty is the transition from childhood to adulthood, characterized by biological maturation. Inter-individual and between-gender differences in puberty timing are associated with multiple biological (e.g., increased height, weight, strength, fat mass) and psychosocial (e.g., self-esteem, body image concerns) changes and challenges for adolescents^13–15^. In relation to physical activity involvement, when comparing same-age youth, earlier-maturing boys might benefit from greater height, weight, and strength advantages^16^, which could facilitate continued participation^14,17^. However, earlier-maturing girls are typically less active due to a host of anthropometric and psychological variables including changes in body composition, feelings of self-consciousness^13,16^ and lower self-concept^16,18^. While the steep drop off in girls participation in sport around the time of puberty is often anecdotally described as challenges with developmental changes, associations between puberty timing and physical activity discontinuation from unorganized and individual physical activity contexts are currently unclear^19^.

Age difference between individuals within the same age group might be another factor which could explain variation in physical activity participation during adolescence. To provide developmentally appropriate physical activity experiences^20,21^, youth are often grouped by chronological age. However, those born earlier in the year (e.g., relatively older) have likely been exposed to a greater number of physical and psychological experiences than their relatively younger (i.e., born later in the year) counterparts^22,23^. This might represent a performance-related advantage for relatively older individuals within a same age group. An over-representation of participants from a given birth quartile (e.g., January-March), relative to participants from other birth quartiles (e.g., September-December) among same-age youth has been termed relative age effect^20^. While relative age effects are likely to depend on individual characteristics (e.g., birth date), task (e.g., specific physical activity), and environment (e.g., cultural importance of activity) constraints^23^, relative age effects have been documented in individual and team-based sports^21^, and organized and unorganized contexts^22^. However, this information is derived from mainly sport-specific cross-sectional studies^20,21^.

Examining physical activity-specific discontinuation and physical activity-general discontinuation concurrently will improve our understanding of physical activity involvement during adolescence. Therefore, we aimed to describe gender-specific longitudinal involvement, discontinuation, re-engagement, and uptake rates in various types and contexts of physical activity from the ages of 11 to 17 years, and to predict gender-specific discontinuation from organized, unorganized, individual, and group-based physical activities using puberty timing and relative age.

## Methods

Data from the Monitoring Activities of Teenagers to Comprehend Their Habits (MATCH) study^24^ was used to test the research questions. Briefly, MATCH is an ongoing longitudinal study aimed at understanding physical activity behavior from childhood to early adulthood. At study inception (2011), participants in grades 5 or 6 (ages 10 to 13 years) were recruited from 17 schools in New Brunswick, Canada. Schools were purposely sampled to represent a mix of urban/rural locations and a variation in socioeconomic status. For the first 24 survey cycles, self-reported data were collected three times per year (September, January–February, and May–June) during the school year until the end of high school (2018). Since this analysis is aimed to better understand age-related discontinuation, re-engagement, and uptake rates of physical activities, we excluded grade 5 data so that the start point of analyses was grade 6 for all participants. To ensure accurate descriptions of physical activity discontinuation timing, we also excluded participants with gaps of ≥ 1 year (3 cycles) between consecutive data collection cycles. Ethics approval was obtained from the Université de Sherbrooke ethics committee and the study was conducted in accordance with recognized ethical standards and national/international laws. All participants provided written informed assent, and parents provided written informed consent.

### Measures

#### Physical activity

To capture youths’ leisure-time involvement in physical activity, participants reported all physical activities they took part in outside of gym class at each cycle (every four months) using a checklist of 36 activities. Participants indicated the frequency (i.e., *never, once a month or less, 2–3 times per month, once a week, 2–3 times per week, 4–5 times per week, or almost every day*), and with whom (i.e., *alone, with friends, with parents and/or siblings, or with an organized group or team*) they most often participated in each activity. We only considered regular physical activities (e.g., at least once a week) to avoid counting spurious activities^25^. In addition, indoor and outdoor chores were excluded since they can be viewed as nonleisure/nonvolitional^26^. Walking was also excluded because it was reported by nearly all participants at each cycle. A detailed list of physical activities and their categorizations is presented in the Appendix.

Each physical activity was also categorized as *organized* or *unorganized,* using a validated method for the MATCH study^27^. Briefly, seven physical activities are classified as *unorganized* (home exercises, trampoline, games, skipping rope, weight training, indoor and outdoor chores). The remaining 29 activities are categorized as *unorganized* if participants reported taking part in the activity alone, with friends, or with parents and/or siblings. Alternatively, if participants reported involvement with an organized group or team, the activity was categorized as *organized*.

Using previously published definitions from the MATCH study^28^, each physical activity was also categorized as *individual* or *group*-based. Whereas 24 activities are always categorized as *individual*, 12 activities (ice hockey, street hockey, ringette, soccer, Canadian football, basketball, baseball, volleyball, handball, dance, ball games, or games) could be categorized as *group*-based if participants reported involvement with an organized group or team, with friends, or with parents and/or siblings. Otherwise, if participants reported participation alone, the activity was classified as *individual.*

#### Predictors of physical activity discontinuation

Puberty timing was categorized as *early-maturation, on-time-maturation,* or* late-maturation,* based on participant self-report using the pubertal development scale (PDS)^29^. All participants self-reported body hair growth using a 4-item Likert scale (*not yet started, barely started, definitely started, seems completed*). Using the same Likert scale, girls answered questions about breast size, whereas boys responded to questions about voice deepening and facial hair growth. Girls were also questioned whether menstruation had started (yes/no). Scores for each question were summated by cycle and PDS means and standard deviation (SD) were computed for girls and boys separately. Then, as was done previously^30^, we classified participants as *early-maturation* if their individual PDS score was one SD higher than the age- and gender-specific PDS mean, *late-maturation* if their individual PDS score was one SD lower than the age- and gender-specific PDS mean, or *on-time-maturation* for participants whose PDS score were within one SD of the age- and gender-specific PDS mean. The PDS demonstrates criterion validity with intraclass correlations (ICC) between physician assessment and self-rating [ICC (95% CI) 0.75 (0.72–0.85)] for girls and [0.72 (0.55–0.81)] for boys and has Cronbach’s α internal consistency coefficients for self-rating of 0.93 for girls and 0.91 for boys^31^.

To align with the majority of sport birthdate cut-offs, relative age was computed by classifying participants into *Birth quartiles* (i.e., Q1: January–March; Q2: April–June; Q3: July–September; and Q4: October–December) based on their birthdate^20,21^. Participants might take part in more than one activity with different birthdate cut-offs for registration. For example, in the province of New Brunswick, birthdate cut-offs for ice hockey (December 31st) are different than those for golf (August 1st) but participating in one activity does not preclude involvement in the other. Therefore, to understand the influence of activity type, sensitivity analyses (results not shown) were conducted where activities with birthdate cut-offs different than December 31 were excluded (e.g., ice skating, golf, and swimming). Since similar results were obtained in both sets of analyses, the current analysis includes all activities regardless of registration cut-off date.

### Data analysis

We calculated the proportion of participants reporting involvement in each physical activity and organized, unorganized, individual, and group-based activities at each grade. We used the PROC LOGISTIC procedure in SAS to assess linear and quadratic trends in these proportions using logistic regressions. Specifically, we used participation (yes/no) in each activity as the outcome and tested age as a linear term. In a separate set of models, age was included both as a linear and quadratic term.

To describe discontinuation in each physical activity, and in organized, unorganized, individual, and group-based activities from grade 7 to grade 12, we determined the number of months that participants in a given physical activity in grade 6 (those who reported the activity at least once per week) continued to report participation in that activity. For each physical activity, we considered discontinuation to have occurred once participation in the given physical activity was not reported for at least 1 year (up to 3 consecutive data collection cycles) after grade 6. For organized, unorganized, individual, and group-based activities, we considered discontinuation to have occurred once participants no longer reported any activity classified into these groupings for at least 1 year after grade 6. Discontinuation rates were calculated with Poisson regressions and are expressed as incidence per 1000 person-month of follow-up with 95% confidence intervals using the PROC GENMOD procedure in SAS. To visualize discontinuation rates for each physical activity, we also produced a timeline indicating the months since baseline when most (i.e., ≥ 50%) participants discontinued participation.

Among participants who discontinued a given physical activity, re-engagement was calculated as the proportion of participants who reported involvement in that same activity at least once per week after an interruption of over one year. Finally, among those who were not involved in a physical activity in grade 6, uptake was calculated as the percentage of participants who started reporting participation in that physical activity in subsequent grades.

To assess puberty timing and relative age as predictors of discontinuation from organized, unorganized, individual, and group-based physical activities, we estimated gender-stratified bivariate Cox proportional hazard models using the “survival” package in R^32^. Discontinuation was considered to have occurred when an individual had a one year or more interruption in meeting the definition of participating in organized, unorganized, individual, or group-based activities. Time until discontinuation was computed as the number of months from the data collection dates between first involvement in a given physical activity type in grade 6 until the last time it was reported before discontinuation. Models were constructed separately for puberty timing and relative age. Puberty was treated as a time-dependent variable since pubertal status relative to peers might change across school grades whereas birth quartile was considered as time-invariant. Investigation of the proportional hazard assumption were conducted by visual inspection of Schoenfeld residuals and hypothesis testing of whether the effect of exposure differed over time. Analyses were conducted in SAS 9.4 (Cary, NC, USA) and in R version 4.2.1.

## Results

Grade 6 physical activity participation data were available for 781 participants (84% of total sample; 57% girls) and were retained for analyses. These participants took part in a median (interquartile range) of 14 (8–20) data collection cycles. Participants were, on average, 11.5 (0.4 SD) years old at study onset and 17.4 (0.3) at study end. In grade 6, most participants (56.5%) had on-time puberty, whereas 14.2% were early-maturing and 29.3% were late-maturing. Twenty-five percent of participants were born between January and March (Q1), 27% between April and June (Q2), 22% between July and September (Q3), and 25% between October and December (Q4). There were no differences in participants’ gender between those who took part in < 5, < 10, or ≥ 15 cycles.

### Prevalence of physical activity participation by school grade

**Figure 1:**
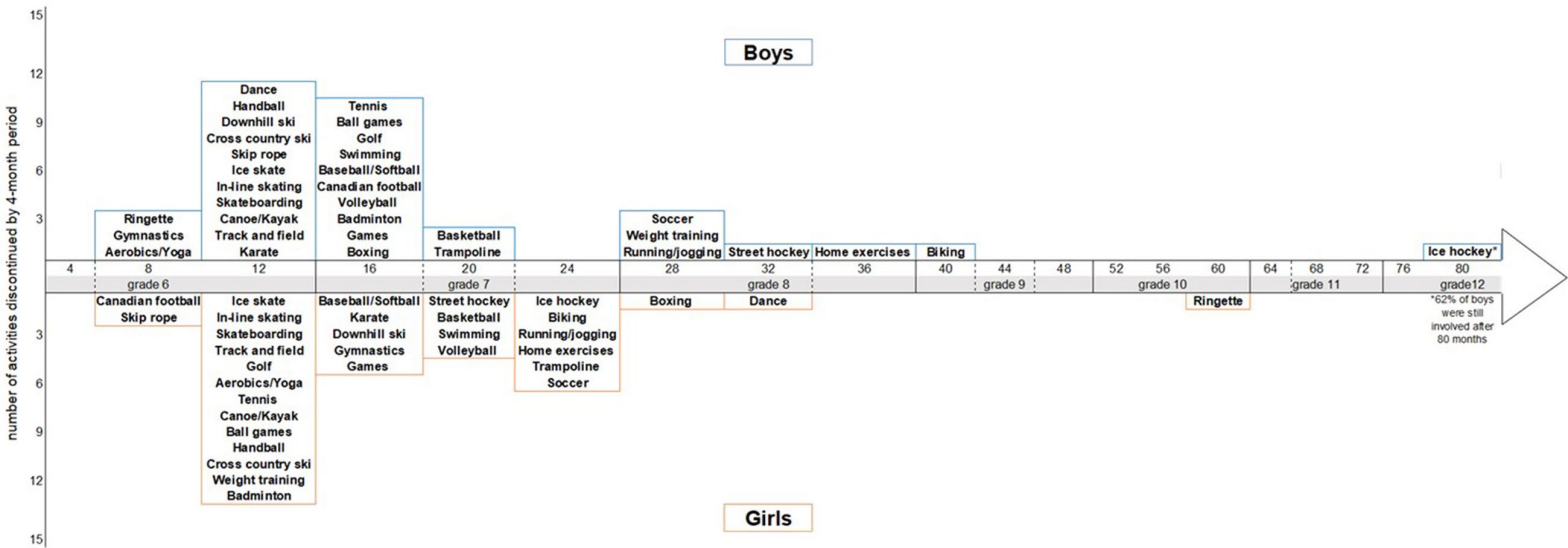
Number of months at which the probability of discontinuing partcicipation in specific physical activities reaches 50%.

Nearly all participants took part in at least one unorganized activity or individual activity in grade 6 (Table 1). In grade 6, the three most frequently reported activities among girls were bicycling (62%), games (chase, tag, hide and seek) (56%), and jogging/running (55%). For boys, the top activities were bicycling (78%), jogging/running (56%), home exercises (pushups, sit-ups) and soccer (54%, each). In grade 12, the top activities for girls were home exercises (46%), jogging/running (31%), and weight training (26%), whereas the top activities for boys were weight training (46%), home exercises (42%), and jogging/running (34%). Linear trends suggest that participation in most specific physical activities declined for both genders (Table 1). However, the proportion of boys and girls reporting aerobics and girls reporting home exercises remained stable from grades 6 to 12. The rates of decline in participation changed (significant quadratic trend) for unorganized activities, badminton, trampoline, and volleyball in both girls and boys. For most physical activities, the point at which the probability of discontinuing participation reached 50% occurred in the first years of follow up (Fig. 1).

**Table 1:** Percentage of participation in specific types of physical activities from grades 6 to 12 in the MATCH study (2011–2018).

	Girls											Boys								
	Grade	6		7	8	9	10	11	12			6	7	8	9	10	11	12		
	n	440		404	368	273	234	213	192	*p*^a^	*p*^b^	341	313	270	204	177	154	131	*p*^a^	*p*^b^
Context																				
Organized		83		82	79	73	69	61	54	** < 0.0001**	0.5167	85	78	77	71	68	61	58	** < 0.0001**	0.3732
Unorganized		97		95	90	80	83	74	70	** < 0.0001**	**0.0008**	99	96	96	90	84	81	80	** < 0.0001**	**0.0346**
Format																				
Group		91		87	81	67	64	54	48	** < 0.0001**	**0.0229**	90	81	80	69	65	61	51	** < 0.0001**	0.1633
Individual		97		96	91	85	86	76	69	** < 0.0001**	**0.0135**	98	96	96	90	87	82	80	** < 0.0001**	0.2226
Type																				
Cross-country skiing		9		9	6	4	3	3	1	** < 0.0001**	**0.0406**	9	8	9	6	7	3	2	**0.0012**	0.1391
Aerobics, yoga, exercise class		20		19	26	27	25	23	20	0.1745	0.0592	10	9	10	9	13	8	9	0.7144	0.7548
Badminton		22		22	15	12	6	3	2	** < 0.0001**	**0.0118**	32	32	23	23	17	6	4	** < 0.0001**	**0.0036**
Ball games (dodge ball, kickball, catch)		34		24	16	6	6	2	3	** < 0.0001**	0.9751	42	34	24	13	11	5	2	** < 0.0001**	0.7705
Baseball		8		9	9	5	4	3	3	**0.0004**	0.1012	14	16	17	7	8	6	5	** < 0.0001**	0.1659
Basketball		15		14	14	7	6	1	1	** < 0.0001**	** < 0.0001**	27	22	21	12	12	12	6	** < 0.0001**	0.3725
Bicycling		62		58	39	32	26	18	11	** < 0.0001**	0.3900	78	80	77	54	50	35	18	** < 0.0001**	**0.0017**
Boxing,wrestling		2		1	1	2	2	0.5	1	0.3485	0.5078	5	4	5	4	7	3	2	0.3973	0.0988
Dance		54		44	37	32	26	22	21	** < 0.0001**	0.0666	8	7	6	5	6	2	3	**0.0047**	0.3792
Downhill skiing		13		11	7	7	5	2	0.5	** < 0.0001**	0.0823	20	12	14	14	15	14	11	0.0846	0.3541
Canadian football		1		2	1	4	0.9	0	0.5	0.5257	**0.0338**	12	11	9	12	12	6	7	**0.0376**	0.3703
Games (chase, tag, hide and seek)		56		43	28	10	8	6	6	**0.0150**	**0.0429**	52	33	24	12	10	6	2	**0.0028**	0.8411
Golf		5		7	4	3	2	2	2	**0.0004**	0.4405	12	16	11	12	9	8	3	**0.0004**	**0.0359**
Gymnastics		26		22	18	13	7	5	4	** < 0.0001**	0.0503	7	4	7	6	3	3	0.8	**0.0039**	0.1271
Handball		15		6	4	0.7	2	0	0.5	** < 0.0001**	0.0997	16	6	6	4	4	1	0	** < 0.0001**	0.6766
Home exercises (push-ups, sit-ups)		48		51	51	54	53	51	46	0.4949	0.0897	54	56	51	52	45	42	42	**0.0011**	0.6966
Ice Skating		24		21	13	7	11	7	6	** < 0.0001**	0.1730	24	17	16	15	8	5	5	** < 0.0001**	0.3947
Ice Hockey		6		6	7	7	9	8	9	0.1088	0.9557	35	36	34	32	27	27	27	**0.0013**	0.9759
Jogging/running		55		52	52	49	49	37	31	** < 0.0001**	**0.0030**	56	51	51	49	44	34	34	** < 0.0001**	0.1037
Karate		6		5	4	2	3	0.5	0.5	** < 0.0001**	0.4553	18	9	13	10	10	5	6	**0.0004**	0.9431
Canoe, Kayak		6		6	4	3	4	4	4	0.0660	0.2034	6	8	4	7	6	3	0	**0.0063**	**0.0220**
In-line skating		13		8	3	1	3	0.9	0.5	** < 0.0001**	0.7918	10	7	9	9	4	0.6	0	** < 0.0001**	**0.0051**
Ringette		12		9	9	7	9	5	4	**0.0004**	0.5444	3	1	4	2	2	0.6	0.8	0.2570	0.2767
Skateboarding		13		8	3	0.7	3	1	0.5	** < 0.0001**	0.8689	10	8	10	9	5	1	0	** < 0.0001**	**0.0023**
Street/floor hockey		3		3	2	5	3	3	3	0.5306	0.5984	26	20	17	15	11	11	6	** < 0.0001**	0.9767
Skip rope		30		13	8	6	5	1	3	** < 0.0001**	0.1044	9	8	10	8	9	10	2	0.4391	0.2310
Soccer		42		40	30	18	17	11	9	** < 0.0001**	0.2631	54	47	43	27	21	17	15	** < 0.0001**	0.2737
Swimming		42		43	29	16	17	15	9	** < 0.0001**	0.8053	31	30	24	20	9	6	10	** < 0.0001**	0.1628
Tennis		7		8	5	3	3	3	2	** < 0.0001**	0.5230	15	12	14	11	7	5	2	** < 0.0001**	**0.0472**
Track and field		16		16	11	7	6	4	2	** < 0.0001**	0.1353	23	19	16	14	8	6	8	** < 0.0001**	0.4800
Trampoline		51		49	35	18	12	5	2	** < 0.0001**	** < 0.0001**	40	35	27	18	13	5	4	** < 0.0001**	**0.0500**
Volleyball		31		38	34	23	16	15	11	** < 0.0001**	** < 0.0001**	14	17	20	16	14	10	7	0.2926	**0.0055**
Weight training		10		12	13	19	22	31	26	** < 0.0001**	0.2034	30	32	38	45	48	49	46	** < 0.0001**	0.1102

^a^*p*-value for statistical significance of the linear time variable in the logistic regression model.

^b^*p*-value for statistical significance of the quadratic time variable in the logistic regression model;

### Discontinuation of physical activity

**Figure 2:**
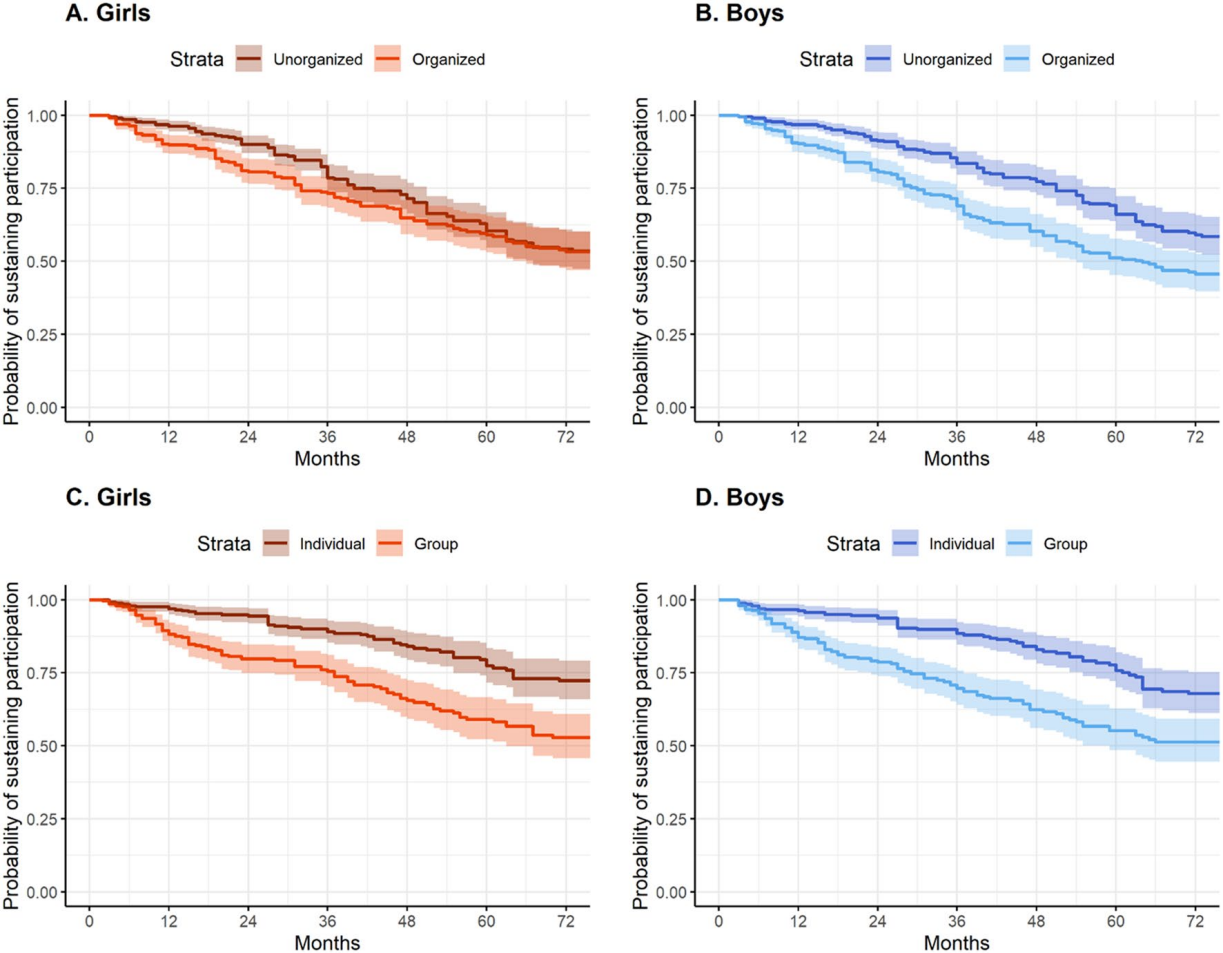
Probability of sustaining participation in organized, unorganized, team, and individual activities for girls and boys in the MATCH study.

By grade 12, 59% of girls were still involved in an individual activity, but only 46% had maintained their involvement in team-based activities (Fig. 2A). For boys, 68% maintained participation in individual activities throughout school years, while 51% continued with team-based activities (Fig. 2B). Slightly more than half (54%) of girls remained involved in unorganized and organized activities by grade 12 (Fig. 2C). More boys maintained their involvement in unorganized activities than organized activities (72% vs. 53% respectively in grade 12; Fig. 2D)).

Among activities with at least 50 participants in grade 6, the physical activity most likely to be sustained for girls was ringette (incidence rate per 1000 person-month follow-up (IR), 95% CI 15, 10–22; Table 2), whereas for boys it was ice hockey (IR, CI 6, 5–9).

**Table 2:** Incidence rates (IR) and 95% confidence intervals (CI) per 1000 person-month follow-up of discontinuation among those who reported taking part in the activity in grade 6.

	Girls				Boys			
	Participants taking part (n)	Person-months follow-up (n)	Discontinued (n)	IR^a^ (95% CI)	Participants taking part (n)	Person-months follow-up (n)	Discontinued (n)	IR^a^ (95% CI)
Context								
Organized	365	14,567	122	8 (7–10)	290	11,084	94	8 (7–10)
Unorganized	425	17,571	124	7 (6–8)	336	14,532	56	4 (3–5)
Format								
Group	399	15,092	151	10 (9–12)	307	11,067	104	9 (8–11)
Individual	427	18,129	108	6 (5–7)	334	14,458	64	4 (3–6)
Type								
Cross-country skiing	39	564	29	51 (36–74)	31	445	27	61 (42–88)
Aerobics, yoga, exercise class	89	1129	60	53 (41–68)	33	430	28	65 (45–94)
Badminton	98	1432	71	50 (39–63)	108	2041	81	40 (32–49)
Ball games (dodge ball, kickball, catch)	149	1950	110	56 (47–68)	143	2437	107	44 (36–53)
Baseball	66	1318	45	34 (25–46)	70	1337	51	38 (29–50)
Basketball	110	2191	65	30 (23–46)	137	2704	89	33 (27–41)
Bicycling	272	5962	192	32 (28–37)	265	8034	136	17 (14–20)
Boxing,wrestling	26	426	17	40 (25–64)	54	956	37	39 (28–53)
Dance	239	6512	132	20 (17–24)	28	345	22	64 (42–97)
Downhill skiing	58	1004	40	40 (29–54)	68	1253	45	36 (27–48)
Canadian football	22	209	18	86 (54–137)	91	1691	61	36 (28–46)
Games (chase, tag, hide and seek)	246	4020	181	45 (39–52)	176	2886	137	47 (40–56)
Golf	23	386	17	44 (27–71)	40	797	24	30 (20–45)
Gymnastics	115	2291	75	33 (26–41)	24	258	22	85 (56–130)
Handball	64	738	54	73 (56–96)	53	677	46	68 (51–91)
Home exercises (push-ups, sit-ups)	212	5229	119	23 (19–27)	185	5734	93	16 (13–20)
Ice Skating	104	1734	78	45 (39–52)	81	1187	70	59 (47–75)
Ice Hockey	36	932	19	20 (13–32)	133	5308	34	6 (5–9)
Jogging/running	242	5535	139	25 (21–30)	190	5366	112	21 (17–25)
Karate	28	419	18	43 (27–68)	61	1307	35	27 (19–37)
Canoe, Kayak	28	294	21	71 (47–110)	22	388	19	49 (31–77)
In-line skating	55	708	43	61 (45–82)	34	568	30	53 (37–76)
Ringette	54	1807	27	15 (10–22)	12	115	11	96 (53–173)
Skateboarding	44	727	30	41 (29–59)	83	1891	58	31 (24–40)
Street/floor hockey	48	1015	32	32 (22–45)	159	4265	97	23 (19–28)
Skip rope	132	1441	107	74 (61–90)	31	456	26	57 (39–84)
Soccer	184	4261	116	27 (23–33)	184	4714	111	24 (20–28)
Swimming	185	3769	117	31 (26–37)	106	2069	73	35 (28–44)
Tennis	30	380	24	63 (42–94)	50	1045	37	35 (26–49)
Track and field	72	1103	58	53 (41–68)	79	1266	66	52 (41–66)
Trampoline	225	4862	165	34 (29–40)	137	2587	100	39 (32–47)
Volleyball	137	3602	95	26 (22–32)	49	1358	31	23 (16–32)
Weight training	42	668	34	51 (36–71)	102	2880	53	18 (14–24)

Discontinuation was considered to have occurred when an individual had a one year or more interruption in meeting the definition of participating in organized, unorganized, individual, or group-based activities.

^a^Incidence Rate (IR) per 1000 person-month follow-up.

### Re-engagement and uptake of physical activities

For girls and boys, the three activity types most likely to be re-engaged after discontinuation were home exercises, weight training, and jogging/running (55%, 36%, 38% [girls]; 43%, 40%, 58% [boys]), respectively; Table 3). Otherwise, less than 1 in 4 participants re-engaged in activities after discontinuation.

**Table 3:** Percentage of participants who re-engaged in each activity after discontinuing for ≥ 1 year.

	Girls			Boys		
	Discontinued (n)	Re-engaged (n)	(%) Re-engagement	Discontinued (n)	Re-engaged (n)	(%) Re-engagement
Context						
Organized	122	31	25	91	28	31
Unorganized	124	70	57	56	26	46
Format						
Group	151	34	23	104	30	29
Individual	108	57	53	64	35	55
Type						
Cross-country skiing	29	3	10	27	4	15
Aerobics, yoga, exercise class	60	16	27	28	6	21
Badminton	71	6	9	81	10	12
Ball games (dodge ball, kickball, catch)	110	11	10	107	16	15
Baseball	45	2	4	51	10	20
Basketball	65	2	3	89	22	25
Bicycling	192	30	16	136	31	23
Boxing,wrestling	17	0	0	37	6	16
Dance	132	14	11	22	1	5
Downhill skiing	40	3	8	45	8	18
Canadian ootball	18	0	0	61	11	18
Games (chase, tag, hide and seek)	181	18	10	137	14	10
Golf	17	3	18	24	4	17
Gymnastics	75	3	4	22	1	5
Handball	54	3	6	46	1	2
Home exercises (push-ups, sit-ups)	119	65	55	93	40	43
Ice Skating	78	13	17	70	10	14
Ice Hokcey	19	2	11	34	8	24
Jogging/running	139	50	36	112	45	40
Karate	18	0	0	35	5	14
Canoe, Kayak	21	3	14	19	0	0
In-line skating	43	0	0	30	0	0
Ringette	27	3	11	11	2	18
Skateboarding	30	3	10	58	5	9
Street/floor hockey	32	3	9	97	16	17
Skip rope	107	10	9	26	4	15
Soccer	116	16	14	111	12	11
Swimming	117	13	11	73	11	15
Tennis	24	1	4	37	1	3
Track and field	58	4	7	66	12	18
Trampoline	165	12	7	100	15	15
Volleyball	95	5	5	31	5	16
Weight training	34	13	38	53	24	45

Discontinuation was considered to have occurred when an individual had a one year or more interruption in meeting the definition of participating in organized, unorganized, individual, or group-based activities.

The list of activities most likely to be initiated after grade 6 is similar to those with the highest level of re-engagement. For both girls and boys, bicycling, home exercises, jogging/running and weight training were activities most frequently associated with an uptake after grade 6 (51%, 60%, 62%, 31% [girls]; 62%, 47%, 52%, 49% [boys], respectively; Table 4).

**Table 4:** Percentage of uptake among participants who did not report the activity in grade 6.

	Girls			Boys		
	(n) not reporting activity in grade 6	Uptake (n)	Uptake (%)	(n) not reporting activity in grade 6	Uptake (n)	Uptake (%)
Context						
Organized	75	42	56	51	24	47
Unorganized	15	10	67	5	2	40
Format						
Group	41	24	59	34	18	53
Individual	13	9	69	7	4	57
Type						
Cross-country skiing	401	39	10	310	41	13
Aerobics, yoga, exercise class	351	142	41	308	63	21
Badminton	342	85	25	233	72	31
Ball games (dodge ball, kickball, catch)	291	62	21	198	51	26
Baseball	374	41	11	271	75	28
Basketball	330	71	22	204	77	38
Bicycling	168	85	51	76	47	62
Boxing,wrestling	414	36	9	287	48	17
Dance	201	57	28	313	38	12
Downhill skiing	382	32	8	273	46	17
Canadian football	418	51	12	250	60	24
Games (chase, tag, hide and seek)	194	76	39	165	34	21
Golf	417	32	8	301	56	19
Gymnastics	325	55	17	317	33	10
Handball	376	19	5	288	27	9
Home exercises (push-ups, sit-ups)	228	137	60	156	73	47
Ice Skating	336	65	19	260	49	19
Ice Hokcey	404	37	9	208	37	18
Jogging/running	198	123	62	151	63	42
Karate	412	23	6	280	22	8
Canoe, Kayak	412	43	10	319	34	11
In-line skating	385	26	7	307	31	10
Ringette	386	15	4	329	26	8
Skateboarding	396	83	21	258	81	31
Street/floor hockey	392	46	12	182	33	18
Skip rope	308	40	13	310	42	14
Soccer	256	74	29	157	56	36
Swimming	255	79	31	235	68	29
Tennis	410	44	11	291	43	15
Track and field	368	68	19	262	56	21
Trampoline	215	72	34	204	53	26
Volleyball	303	95	31	292	57	20
Weight training	398	124	31	239	117	49

### Association between puberty timing and relative age and discontinuation from various physical activities

Girls reporting *early-maturation* were more likely to discontinue organized sports than girls with *on-time-maturation* status (Hazard Ratio (HR), 95% confidence intervals (CI) 1.68 (1.05–2.69); Table 5). Puberty timing was not associated with dropout in boys.

**Table 5:** Hazard ratios and 95 confidence intervals for dropout of organized, unorganized, team, and individual physical activities for girls and boys according to puberty timing^‡^.

	Early-maturation	On-time-maturation	Late-maturation
Girls			
Organized (n = 367)	**1.68 (1.05**–**2.69)**	1.00	1.40 (0.89–2.21)
Unorganized (n = 425)	1.15 (0.68–1.95)	1.00	0.80 (0.47–1.35)
Group (n = 378)	1.04 (0.65–1.67)	1.00	1.36 (0.91–2.03)
Individual (n = 432)	1.51 (0.87–2.59)	1.00	0.66 (0.36–1.22)
Boys			
Organized (n = 290)	1.04 (0.61–1.76)	1.00	0.65 (0.34–1.23)
Unorganized (n = 336)	0.77 (0.36–1.66)	1.00	0.72 (0.32–1.61)
Group (n = 308)	0.81 (0.47–1.40)	1.00	1. 17 (0.71–1.91)
Individual (n = 334)	1.27 (0.69–2.32)	1.00	0.37 (0.13–1.04)

^‡^Puberty timing is treated as a time-dependent variable in the models; bold represents statistical significance at *p* < 0.05.

Birth quartile was not associated with physical activity discontinuation in girls (Table 6). Compared to boys born in Q4, boys born in Q2 were less likely to discontinue from organized (HR [95% CI] 0.41 [0.23–0.74]), unorganized (HR [95% CI] 0.33 [0.16–0.70]), individual (HR [95% CI] 0.58 [0.34–0.98]), and group-based (HR [95% CI] 0.46 [0.23–0.91]) physical activities. Being born in Q3 was also associated with a decreased likelihood of discontinuation from unorganized physical activity (HR [95% CI] 0.41 [0.19 to 0.88]).

**Table 6:** Hazard ratios and 95 confidence intervals for dropout of organized, unorganized, team, and individual physical activities for girls and boys according to birth quartile.

	Q1	Q2	Q3	Q4
Girls				
Organized (n = 367) (Q1_n_ = 90; Q2_n_ = 104; Q3_n_ = 81 Q4_n_ = 92)	0.83 (0.51–1.38)	0.73 (0.45–1.18)	1.07 (0.65–1.76)	1.00
Unorganized (n = 425) (Q1_n_ = 104; Q2_n_ = 104; Q3_n_ = 97 Q4_n_ = 113)	0.73 (0.44–1.21)	0.89 (0.56–1.40)	0.83 (0.50–1.36)	1.00
Group (n = 378) (Q1_n_ = 93; Q2_n_ = 104; Q3_n_ = 85 Q4_n_ = 96)	1.08 (0.69–1.69)	0.81 (0.52–1.27)	1.35 (0.86–2.12)	1.00
Individual (n = 432) (Q1_n_ = 107; Q2_n_ = 114; Q3_n_ = 98 Q4_n_ = 113)	0.69 (0.40–1.20)	0.85 (0.52–1.38)	0.89 (0.52–1.51)	1.00
Boys				
Organized (n = 290) (Q1_n_ = 80; Q2_n_ = 78; Q3_n_ = 62 Q4_n_ = 70)	0.67 (0.39–1.18)	**0.41** **(0.23**–**0.74)**	0.81 (0.47–1.39)	1.00
Unorganized (n = 336) (Q1_n_ = 89; Q2_n_ = 93; Q3_n_ = 75 Q4_n_ = 79)	0.53 (0.27–1.03)	**0.33** **(0.16**–**0.70)**	**0.41** **(0.19**–**0.88)**	1.00
Group (n = 308) (Q1_n_ = 82; Q2_n_ = 82; Q3_n_ = 73 Q4_n_ = 71)	0.62 (0.37–1.05)	**0.58** **(0.34**–**0.98)**	0.69 (0.41–1.18)	1.00
Individual (n = 334) (Q1_n_ = 88; Q2_n_ = 93; Q3_n_ = 74 Q4_n_ = 79)	0.67 (0.36–1.25)	**0.46** **(0.23**–**0.91)**	0.52 (0.25–1.06)	1.00

Q1, January–March; Q2, April–June; Q3, July–September; Q4, October–December; bold represents statistical significance at p < 0.05.

## Discussion

In this study, we documented that participation in most physical activities decreased from ages 11 to 17 years. Our results also highlight that although most group-based and organized activities were not re-engaged after discontinuation, re-engagement was considerably more likely for individual-based and unorganized activities. This said, despite high rates of dropout from most specific physical activities during adolescence, many participants still maintained involvement in some individual and unorganized physical activity by the end of high school. Some activities had relatively higher likelihoods of being sustained throughout adolescence than others. We also found that earlier-maturing girls had a higher risk of discontinuing organized activities than other girls, and that boys born between April and June had a lower risk of discontinuing organized, unorganized, individual, and group-based activities.

Participation in most physical activities included in this study declined during adolescence. The similarity between the results of the current study and those conducted a decade ago suggest that teenage physical activity participation patterns have remained largely similar^2,3^. In line with the theory of biological regulation^33^, it is possible that declines in physical activity during adolescence are simply a natural part of aging. Despite this, our results clearly indicate that individual activities are generally sustained longer than group-based activities and that unorganized activities are more likely to be sustained than organized activities. Whereas previous studies also suggested that individual activities have a higher likelihood of being sustained longer than group-based activities^2^, this is the first study to describe sustainability in organized and unorganized activities during adolescence. These results highlight the importance of exposing and facilitating access to individual and unorganized physical activity for adolescents. Most specifically, our results point to fitness-focused activities (e.g., home exercises, weight training, running/jogging) as those with some of the highest potential for uptake, re-engagement, and maintenance during adolescence. Fitness-focused activities are also among the most frequently reported by active young adults^34^, which raises the hypothesis that they could contribute to lifelong physical activity participation if started during adolescence. These activities may display the most promise for long-term participation because they are associated with relatively few organizational barriers and are low-cost^7^, which also makes them appealing as targets for wide-reaching interventions.

Our observation that early maturation in girls predicts discontinuation from organized physical activities is in line with previous studies reporting an association between sport participation and puberty timing^35,36^. It is possible that girls’ earlier changes in physical factors, including increased fat mass, breast development and widening of the hips^37^ is accompanied by changes in psychosocial factors such as self-consciousness^13,16^, lower self-concept^18^ or teasing^38^ leading to increased likelihood of dropout. Such changes modulate girls’ idea of ideal body shapes and sizes and, therefore, often generate dissatisfaction with their bodies^39^. In turn, sub-optimal self-perceived body image is linked to lower physical activity levels, potentially owing to the avoidance of situations in which girls may be judged for their appearance^39^. In this respect, it may be advisable to promote physical activities that involve fewer opportunities for physical comparisons with peers (i.e., individual and unorganized physical activities) in advance of puberty so that girls can be active without worrying. Consequently, it is important to encourage girls to stay active and overcome self-imposed barriers associated with body image or misconceptions about how they should look during physical activities, as early evidence shows that interventions can improve satisfaction with body-image and increase intentions to engage in physical activity^40^.

In contrast, we found no associations between puberty timing and risk of dropping out of physical activities among boys. This conflicts with results from studies suggesting that boys with late-maturation are more likely to dropout from sports^41–44^. However, previous studies did not account for the impact of uptake of new physical activities following drop out. It is, therefore, possible that puberty-associated physical activity dropout among boys is nullified by engagement in a different activity.

Birth quartile was a predictor of physical activity drop out among boys, but not girls in this study. While relative age effects are typically identified as an increased proportion of participants born in Q1 vs. Q4, some activities display Q2 vs. Q4 differences^20^, similar to results found in this study. However, finding that birth quartile was protective against unorganized physical activity discontinuation was unexpected since unorganized physical activity is usually practiced by individual volition^27^ and therefore would not include a selection process. This suggests that talent selection is not the only driver of relative age effects^23^ and warrants future investigation into correlates of relative age effects and how they might present differently in unorganized physical activity than in organized physical activity contexts. The lack of relative age effects among girls in this study is different than previous findings^21^, but might reflect girls’ lower likelihood to participate in competitive sports involving a selection process.

The application of frequent assessments over a 7-year period provided the unique opportunity to present a comprehensive overview of changes in participation in various physical activities among adolescents. In addition, investigating sustainability in organized, unorganized, individual, and group-based physical activity provided information on domain-specific dropout. These data also allowed describing the contribution of puberty and relative age as predictors of change in physical activity participation. It nevertheless needs to be recognized that all measures were based on self-report and could therefore be associated to some over/under-estimation. Also, some activities classified as individual might have been taken part with others (e.g., swimming). Given our aim of documenting change in participation in different physical activity types during adolescence, the classification scheme of individual and group-based activities precluded the study of social context of participation. Future investigations of physical activity discontinuation that consider the social context of participation is warranted, given the importance of the social environment for the maintenance of physical activity^45^. In addition, since we defined discontinuation as not reporting an activity for at least 1 year, we were unable to capture activities that were discontinued during the final year of the study. We were unable to control for confounding factors such as BMI in associations between puberty timing and physical activity discontinuation, due to data unavailability. Further, although participants retained for the MATCH study were purposely sampled to represent a mix of urban/rural locations and a variation in socioeconomic status within a province, these results may not generalize to other samples from other countries given national and regional cultural differences in physical activity and sport participation. Researchers from other countries and/or areas could use the current methodology to replicate this study in other regions.

In conclusion, this study highlights that although there is a marked decline in participation in most physical activities during adolescence, general participation in physical activity may persist through sustainment of some activities, uptake of new ones and re-engagement in others. Physical activities most likely to be sustained, re-engaged after discontinuation, or initiated during adolescence were mostly unorganized and individual activities. This suggests that these activities have a potential to be carried over into adulthood. Specifically, interventions aimed at fostering individual and unorganized activity participation among adolescents are likely worthwhile since these types of activity are maintained longer and are more likely to be reengaged in than organized and group-based activities.

### Supplementary Information

Supplementary Information.

## Data Availability

The datasets generated during and/or analysed during the current study are not publicly available to insure confidentiality and that any secondary analyses correspond to the objectives of the research project, but are available from mathieu.f.belanger@usherbrooke.ca on reasonable request.

## Abbreviations

CI: Confidence interval
HR: Hazard ratio
IR: Incidence rate
MATCH: Monitoring activities of teenagers to comprehend their habits
PDS: Pubertal development scale
