# Supplementary material for: Puberty timing and relative age as predictors of physical activity discontinuation during adolescence
Source: Sci Rep. 2023 Aug 23;13:13740. doi: 10.1038/s41598-023-40882-3 (PMC10447449; doi:10.1038/s41598-023-40882-3)
Supplement: Supplementary file 1 — Supplementary Information. [file 41598_2023_40882_MOESM1_ESM.docx]

**Puberty timing and relative age as predictors of physical activity discontinuation during adolescence**

François Gallant, Jeff J. Hebert, Véronique Thibault, Saïd Mekari, Catherine M. Sabiston, Mathieu Bélanger

| Appendix. Classification of each activity type into organized, unorganized, group, or individual in the MATCH study | | | | |
| --- | --- | --- | --- | --- |
| **Activity type** | **Organized activity** | **Unorganized activity** | **Group** | **Individual** |
| Street hockey, floor hockey | A | B | C | D |
| Ice hockey | A | B | C | D |
| Ringette | A | B | C | D |
| Ice skating (not for hockey or ringette) | A | B |  | X |
| In-line skating | A | B |  | X |
| Skateboarding or scooter | A | B |  | X |
| Bicycling | A | B |  | X |
| Track and field | A | B |  | X |
| Jogging or running | A | B |  | X |
| Golfing | A | B |  | X |
| Swimming | A | B |  | X |
| Gymnastics | A | B |  | X |
| Aerobics, yoga, exercise class | A | B |  | X |
| Home exercise (push-ups, sits-ups) |  | X |  | X |
| Baseball or softball | A | B | C | D |
| Weight training |  | X |  | X |
| Basketball | A | B | C | D |
| Canadian football | A | B | C | D |
| Soccer | A | B | C | D |
| Volleyball | A | B | C | D |
| Badminton | A | B |  | X |
| Tennis | A | B |  | X |
| Kayak, canoe | A | B |  | X |
| Dance | A | B | C | D |
| Trampoline |  | X |  | X |
| Skipping rope |  | X |  | X |
| Handball or mini handball | A | B | C | D |
| Ball playing (dodge ball, kickball, catch) | A | B | C | D |
| Games (chase, tag, hide and seek) |  | X | C | D |
| Downhill skiing or snowboarding | A | B |  | X |
| Boxing, wrestling | A | B |  | X |
| Karate, judo, tai chi, taekwondo | A | B |  | X |
| Cross-country skiing | A | B |  | X |
| A = classified in this category if most often practiced with an organized group or team; B = classified in this category if most often practiced alone, with siblings, with parents, with friends;  X = always classified as this type of activity;  C = classified in this category if most often practiced with an organized group or team, with siblings, with parents or with friends;  D = classified in this category if most often practiced alone | | | | |
